# Supplementary material for: Improved Reference Genome Annotation of Brassica rapa by Pacific Biosciences RNA Sequencing
Source: Front Plant Sci. 2022 Mar 17;13:841618. doi: 10.3389/fpls.2022.841618 (PMC8968949; doi:10.3389/fpls.2022.841618)
Supplement: Supplementary file 1 [file Table_1.DOCX]

Supplementary Material

## Supplementary Figures


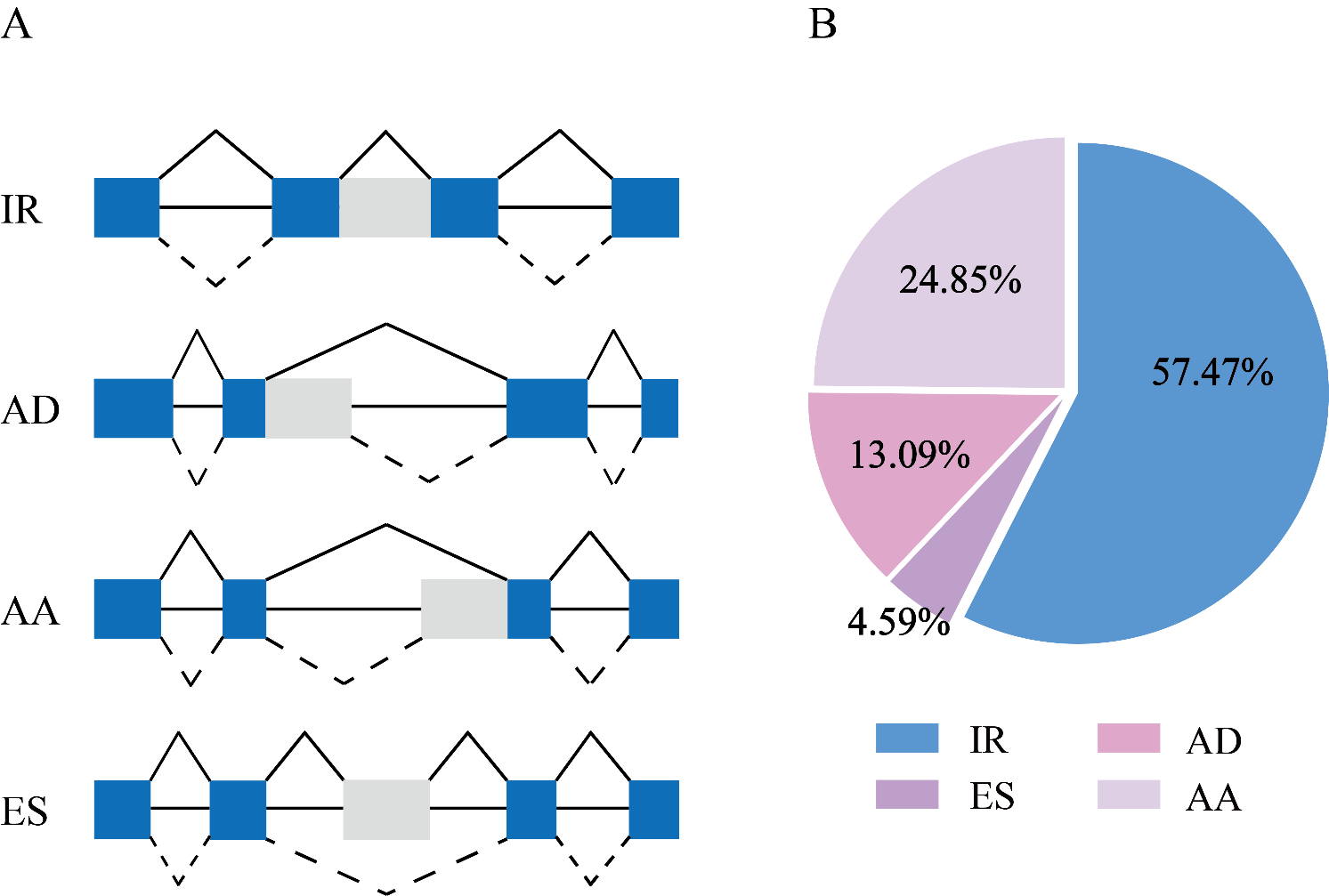


**Figure S1.** (A) Patterns of alternative splicing. The boxes represent exons, introns are represented by black straight lines. (B) Proportions of four AS types classified according to biogenesis.


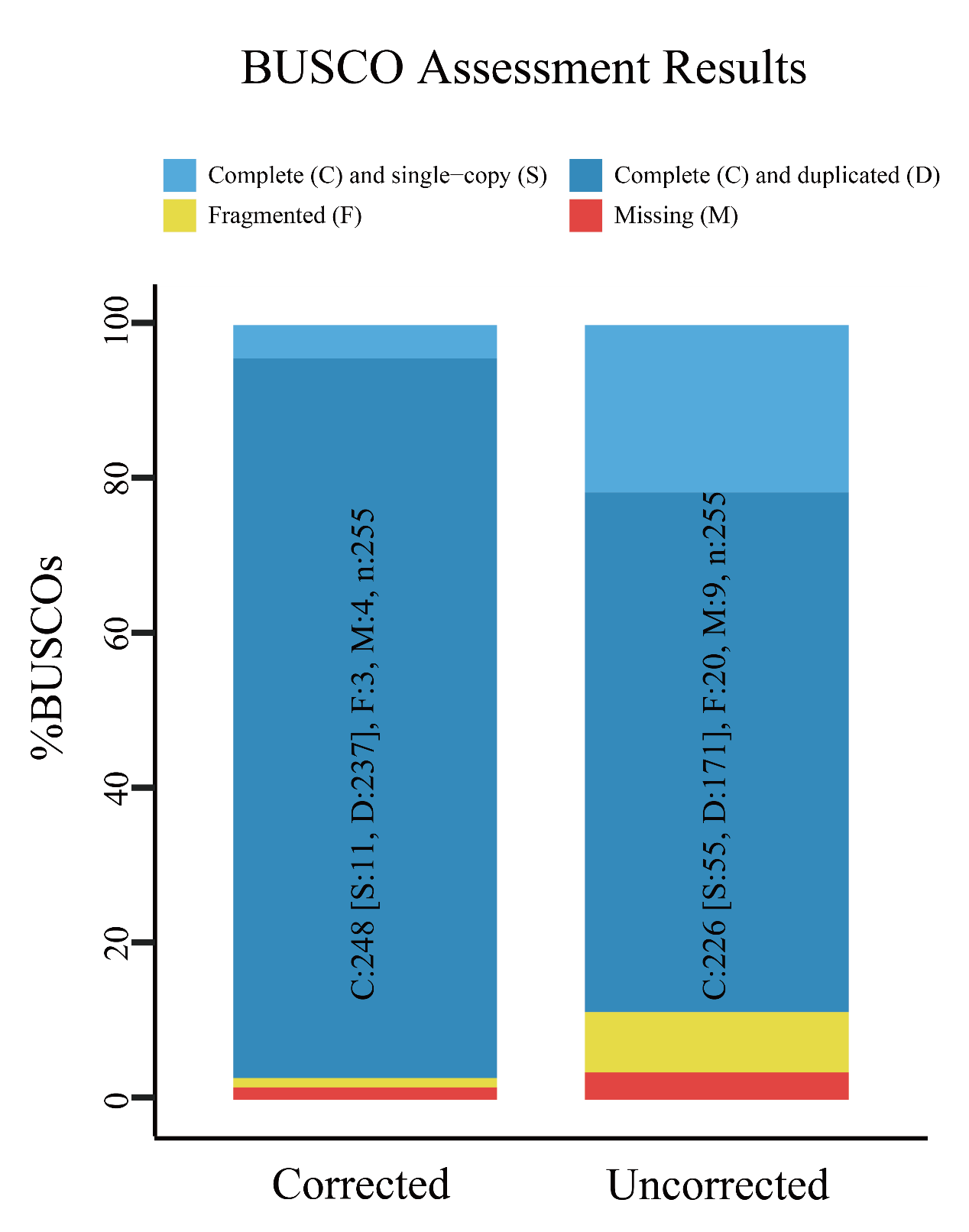


**Figure S2.** BUSCO assessment result of corrected and uncorrected FLNC reads.


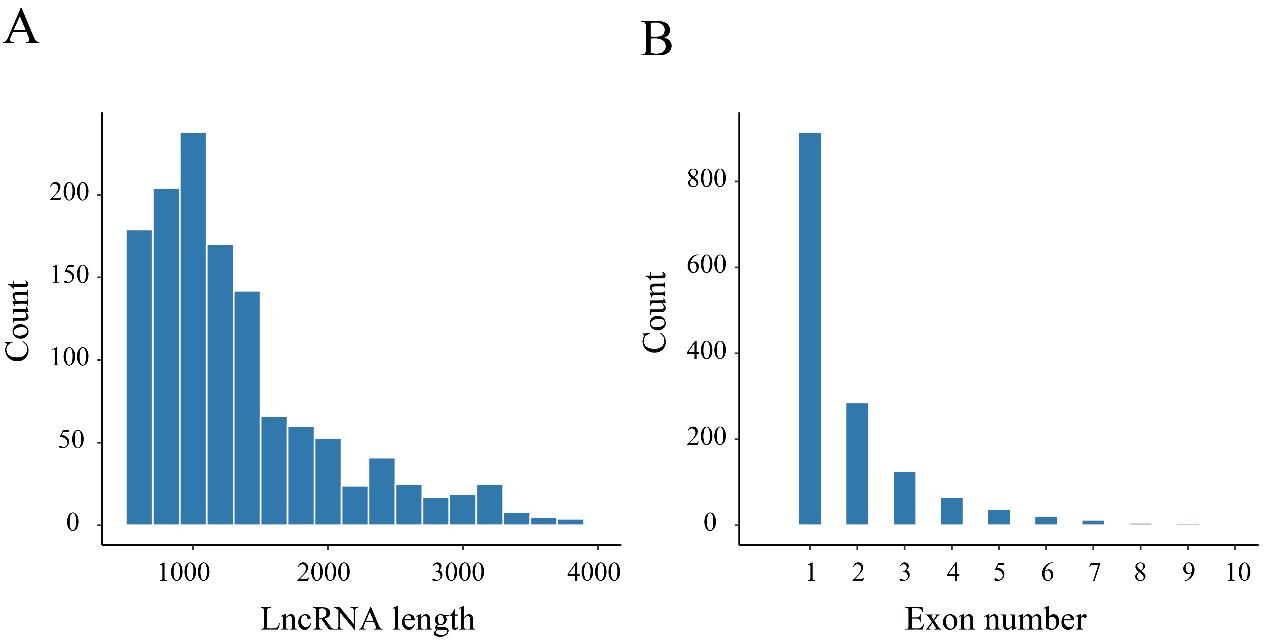


**Figure S3.** (A) and (B) Frequency distribution of length and exon number of lncRNAs.
